# “This bicycle gives me a headache”, a congenital anomaly

**DOI:** 10.1186/1756-0500-6-412

**Published:** 2013-10-14

**Authors:** Hendt P Versteegh, Wout FJ Feitz, Erik J van Lindert, Carlo Marcelis, Ivo de Blaauw

**Affiliations:** 1 Department of Pediatric Surgery, Erasmus MC Sophia Children’s Hospital, Rotterdam, the Netherlands; 2 Pediatric Urology Center, Department of Urology, Radboud University Medical Center, Nijmegen, the Netherlands; 3 Department of Neurosurgery, Radboud University Medical Center, Nijmegen, the Netherlands; 4 Department of Clinical Genetics, Radboud University Medical Center, Nijmegen, the Netherlands; 5 Department of Surgery-Pediatric Surgery, Radboud University Medical Center, Nijmegen, the Netherlands

## Abstract

**Backround:**

The combination of a presacral mass, a sacral bone deformity, and an anorectal malformation are also known as the Currarino triad or Currarino syndrome. The syndrome is associated with a very high rate of severe and intractable constipation and urinary incontinence. However, it can also result in less common complaints and symptoms. Although the syndrome is known since 1981 and the involved genes are clarified to a great extent, the diagnosis may be delayed or missed if unrecognized.

**Case presentation:**

A 24-year old female presented with periodical headaches. She was born with an imperforate anus, absent rectum and colon, double bladder, and sacral defect. Soon after birth she underwent several surgical procedures for anorectal and bladder reconstructions. The patient now came to her pediatric urologist for urinary incontinence and mentioned severe headaches on the side, particularly when riding a bike. Finally, she solved her headache problem by stopping to ride her bicycle.

On physical examination no abnormalities were found except the ileostomy that was present ever since soon after birth and her urinary incontinence. Blood tests showed no abnormalities. Additional MRI showed a large and previously not known anterior meningocele at the level of the sacrum. Surgical treatment consisted of closure of the dura by posterior approach.

**Conclusion:**

In this case report we describe the late discovery with an atypical presentation of an anterior meningocele in a young adult with urinary incontinence, a sacral defect, an anorectal malformation and headaches during bicycle riding. After surgical treatment of our patient the meningocele regressed. Three months after successful surgery she had no complaints and was able to ride a bike again.

## Background

The Currarino syndrome is a congenital syndrome with a classical triad consisting of an anorectal malformation, a sacral defect and a presacral mass or tumor [1]. It was first classically described in three cases by the radiologist Currarino. Since defects on the HLBX9 gene were found to be the cause, more than 350 cases have been described, all with variable expression [2]. The variable expression makes recognition often difficult, particularly in adolescents and adults with little or atypical symptoms [3,4]. With this case report we aim to demonstrate such an atypical presentation and discuss the etiology, management and outcome of patients with the Currarino syndrome.

## Case presentation

### Case

**Figure 1:**
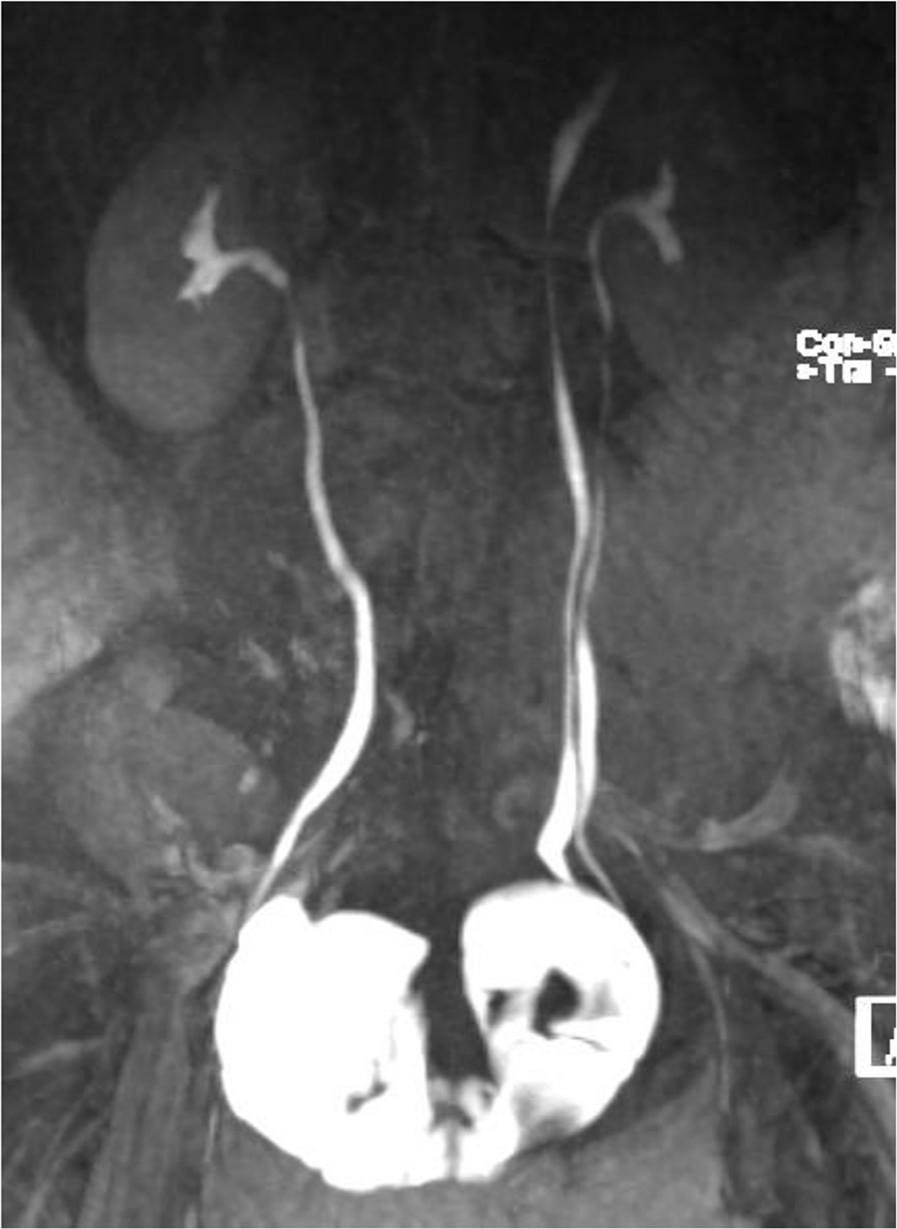
The MRI of our patient showed a double bladder with urinary output of both kidneys to the bladders.

**Figure 2:**
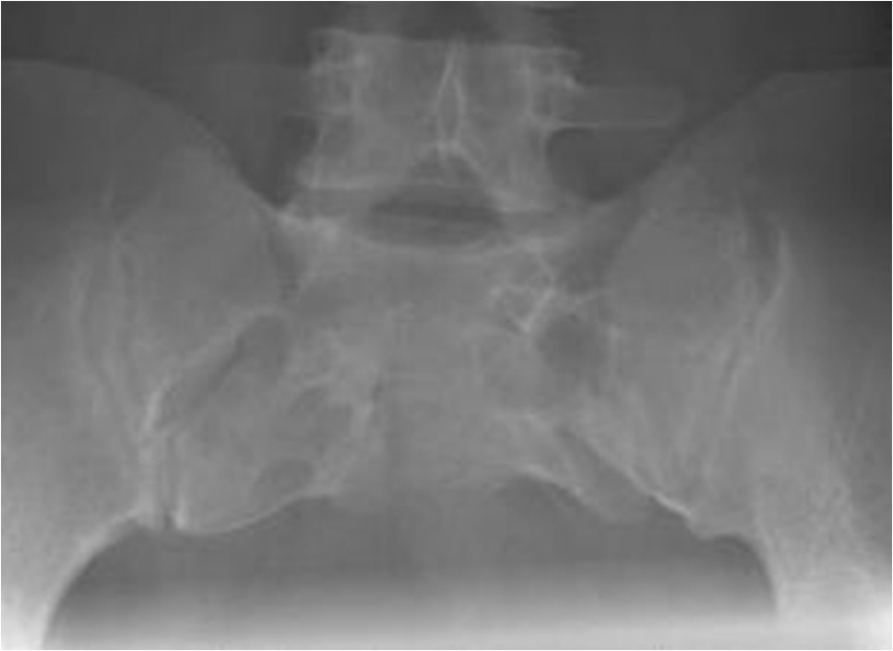
On conventional sacral X-ray a dysgenetic sacrum was seen.

A 24-year old female presented with urinary incontinence and periodical headaches. She was born with an imperforate anus, absent rectum and colon, double bladder (Figure 1), and a dysgenetic sacrum defect (Figure 2). Soon after birth she was given an ileostomy, and later underwent an anorectal and bladder reconstructions. She now came to her pediatric urologist for urinary incontinence and mentioned severe headaches on the side, particularly when riding a bike. She solved her headache problem by stopping to ride her bicycle. Her parents then sold the bicycle since it became useless.

**Figure 3:**
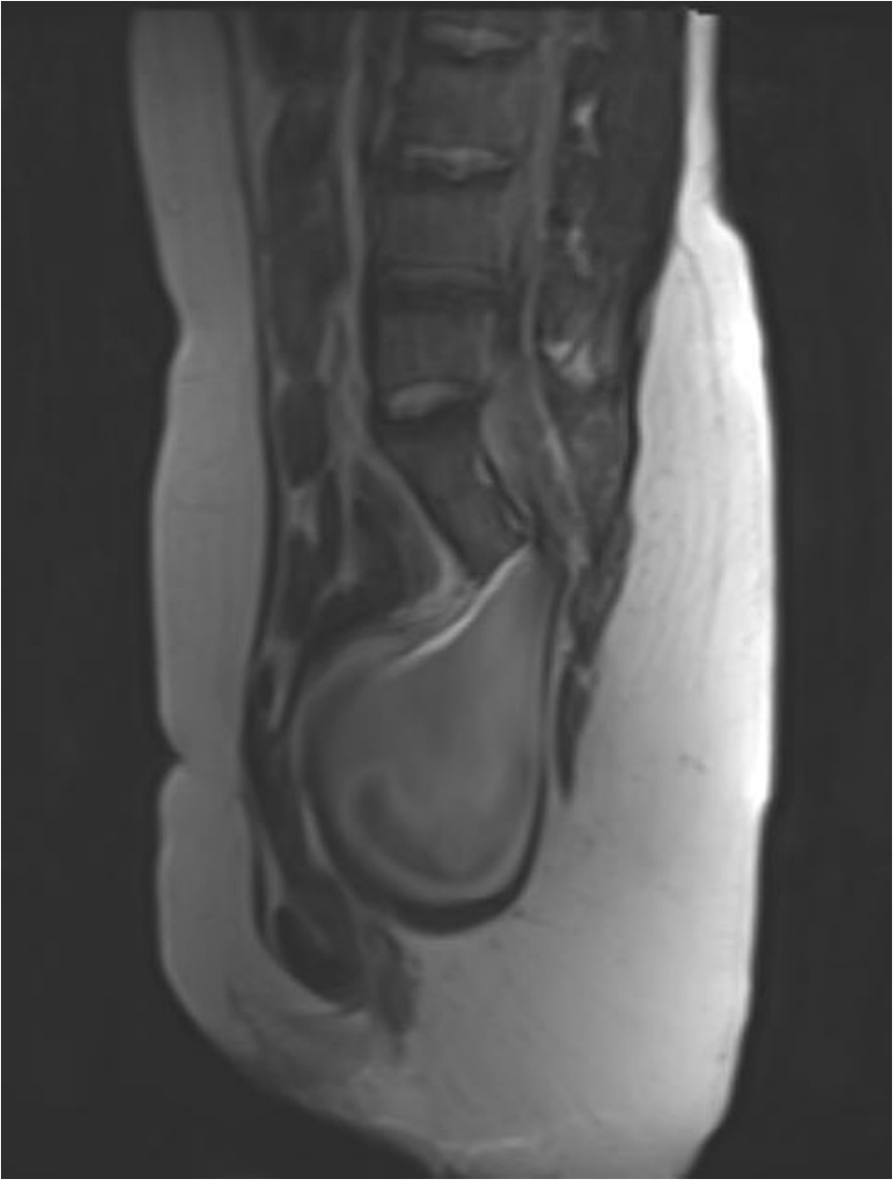
Preoperative MRI showed: agenetic sacrum (from S4 and S5) with an anterior meningocele was seen.

**Figure 4:**
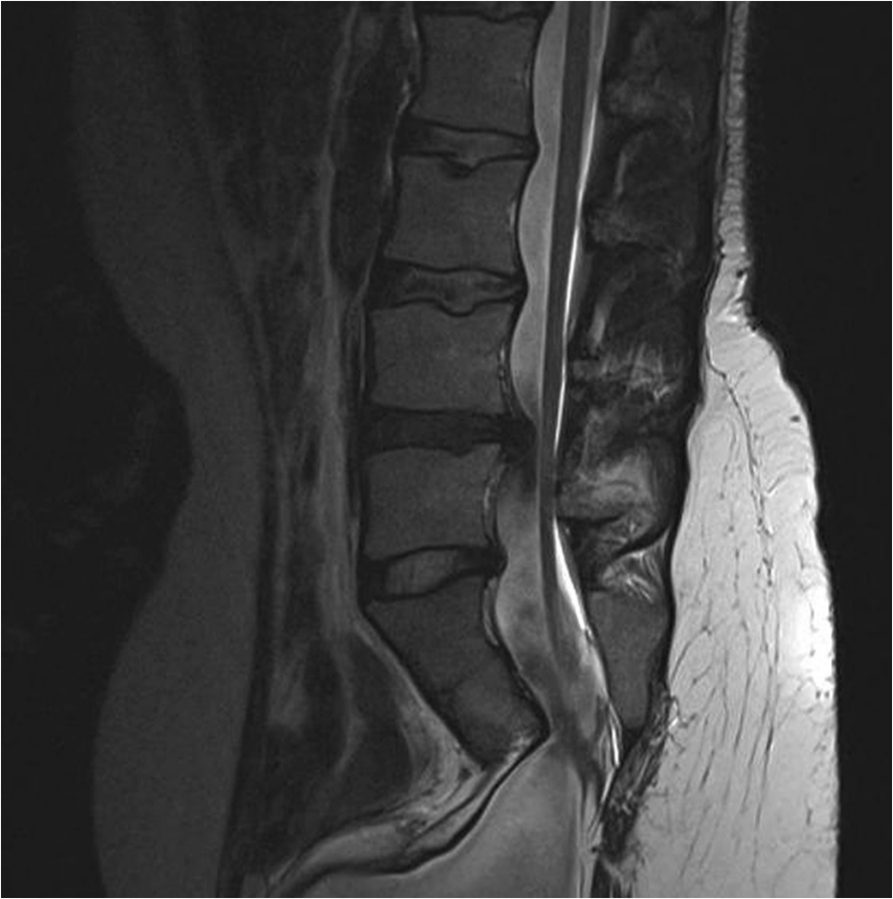
**On the MRI image tethering of the spinal cord was seen at L5-S1 level.** Additonally a herniation of an intervertebral disc is seen (asymptomatic and left untouched at surgery).

Recently she expressed the wish to become dry and additional investigations were scheduled. On physical examination a normal looking woman presented herself with an ileostomy and urinary incontinence without further palpable abnormalities in the abdomen or back. Blood tests showed no abnormalities; particularly alpha foeto-protein and B-HCG were within normal ranges. MRI showed a large and previously not known anterior presacral meningocele (Figure 3) as well as a tethered cord due to a tight filum terminale (Figure 4). She refused testing for defects on the HLXB9 gene on chromosome 7q36. She was referred to a neurosurgeon and treatment of the meningocele consisted of a laminectomy S1-S2, transection of the filum terminale and an intradural closure of the dura towards the meningocele. The meningocele regressed and is expected to further dissolve. Three months after surgery she has no complaints; she received a urinary diversion and bought a new bicycle again.

### Currarino syndrome

The Currarino syndrome is a familiar syndrome with the triad of sacral defects, hindgut malformations and presacral tumors. It has an autosomal dominant inheritance with variable expression and the mutation is on the HLXB9 gene located on chromosome 7q36 [2,5]. The sacral defects vary from a slightly dysplastic sacrum to a complete sacral agenesis (caudal regression). The embryology of the syndrome is still unclear and was initially thought to be a consequence of mal-communication of the endoderm and the neuronal ectoderm [1]. More recently it is suggested that developmental errors in progenitor cells at the region of the caudal eminence are responsible for the abnormality [6,7].

Clinically, the presacral mass in the Currrarino syndrome is a teratoma in approximately 25% of the cases [8]. The complete Currarino triad is rare; this syndrome should be considered even in the presence of a partial phenotype [3,6]. The sacral defects can be mild and go unnoticed. The anorectal malformation can be present but the Currarino syndrome has also been described without an anorectal malformation [9]. Constipation, however, is present in over 95% of the cases, often with an early onset as an infant and often with an intractable character [10]. The wide phenotypic variability requires combined pediatric, neurosurgical, and often urological assessments. The triad is associated with other spinal abnormalities in the majority of patients, including anterior meningoceles in 60% [8]. Anterior meningoceles are congenital lesions of spinal fluid filled sacs communicating with the subarachnoid space [11]. They can give clinical symptoms related to the pressure the cele gives to organs in the pelvic floor region (rectum, bladder, genitals) or to the sacral nerves with subsequent failure of these nerves. Also, pressure on the cele gives increased intracerebral pressure with subsequent headaches.

Anterior meningoceles give symptoms in approximately two-third of the cases. These are abdominal in 70% of the cases, urogenital in 30% of the cases and neurological in 27% of the cases [11]. Beside these effects it may give enteral fistulas resulting in severe infections with life-threatening meningitis [10]. These symptoms appear usually in the second to third decade of life. Anterior meningoceles can further give constipation, abdominal pains, constipation, urinary retention, dysmenorrhea, low back pain, radiation to the legs as first symptoms. However, as in our case other atypical symptoms can be seen as well. Treatment of anterior meningoceles can be conservative if no symptoms are present. However, most anterior meningoceles give symptoms and need surgical treatment because there will be no spontaneous regression. Conservative management is furthermore associated with a 30% mortality rate mostly due to meningitis. Therefore, surgical closure is generally recommended [9,10]. A posterior approach, transsacral or sagittal, is the preferred one because of the lowest complication rate [9,12]. The anterior laparotomy approach still has a high morbidity and mortality (22%) due to infections and fistula formation. Transvaginal and transrectal punctures have also previously been suggested but have a similar high morbidity and mortality.

## Conclusion

This case report illustrates the presentation of an anterior meningocele as part of the Currarino syndrome. The Currarino syndrome can present itself partially and often remains unknown for several years. Severe constipation or mild anorectal malformations combined with any sacral anomaly should make clinicians aware of the possibility of the presence of a Currarino syndrome. MRI is then warranted to rule out any sacral mass or spinal abnormalities and a clinical geneticist should be consulted for further analysis of the syndrome. Our case further demonstrates that atypical symptoms such as periodical headache while riding a bicycle can lead to an unexpected diagnosis.

## Consent

Written informed consent was obtained from the patient for publication of this case report and any accompanying images. A copy of the written consent is available for review by the Editor of this journal.

## Competing interests

All authors declare not to have any conflicts of interest or source of funding, fees or salaries related to the publication of this manuscript.

## Authors’ contributions

WFJF and IdB contributed to conception and design of the study. Data acquisition and analysis was conducted by HPV and IdB. HPV, WFJF, EjvL, CM and IdB were responsible for data interpretation. All authors contributed to drafting of the article or revising of content. All authors gave approval for the final version of the manuscript.
